# Supplementary material for: Identification of two terpenoids from Withania coagulans with predicted multitarget binding affinity: An in vitro and in silico study
Source: PLoS One. 2026 Feb 20;21(2):e0343273. doi: 10.1371/journal.pone.0343273 (PMC12923132; doi:10.1371/journal.pone.0343273)
Supplement: S1 Protocol — (DOCX) [file pone.0343273.s001.docx]

**(Stepwise Protocols S)**

**In Silico Study – Stepwise Protocol**

**1. Protein Preparation**
1.1 Download crystal structures from PDB: OmpF porin (4KR4), HipBST (7AB4), anti-oxidant protein (5U3A), alpha-amylase 2A, KEAP1.
1.2 Open each PDB in BIOVIA Discovery Studio 2021.
1.3 Remove water and heteroatoms via View → Hierarchy → Water + HETATM → Delete.
1.4 Save the cleaned PDB.
1.5 Open the cleaned PDB in Swiss-PdbViewer v4.10.
1.6 Fix missing atoms and residues.
1.7 Save the corrected structure as protein-fix.pdb.

**2. Pharmacological and Drug-Like Properties**
2.1 Retrieve canonical SMILES of ligands from PubChem.
2.2 Input SMILES into SwissADME, pkCSM, and ProTox 3.0.
2.3 Record parameters: TPSA, GI absorption, BBB permeant, CYP2D6 inhibitor, CYP3A4 inhibitor, CYP1A2 inhibitor, Lipinski, Veber, Egan, Log Kp (skin permeation), bioavailability score, water solubility (consensus), GI absorption (human), skin permeability, P-glycoprotein substrate/inhibitor, VDss (human), fraction unbound (human), CNS permeability, CYP2D6/3A4/1A2/2C19/2C9 substrate/inhibitor, total clearance, renal OCT2 substrate.

**3. Ligand Preparation**
3.1 Download ligand structures from PubChem in SDF format.
3.2 Convert SDF to PDB using Open Babel.
3.3 Energy-minimize ligands using steepest descent algorithm with UFF.
3.4 Convert minimized ligands to PDBQT in PyRx 0.8, adding hydrogens, assigning charges, and defining torsional flexibility.

**4. Molecular Docking**
4.1 Load protein-fix.pdb as macromolecule in PyRx.
4.2 Load ligands via Open Babel.
4.3 For redocking, save co-crystallized ligand from original PDB, convert via Open Babel, and dock into the active site.
4.4 Perform docking with exhaustiveness = 8.
4.5 Select ligand conformations interacting with active-site residues.
4.6 Record docking scores (kcal/mol) and analyze interactions in Discovery Studio.

**5. Density Functional Theory (DFT)**
5.1 Open GaussView v5.0.8.
5.2 Load ligand PDB.
5.3 Go to Calculate → Gaussian Calculation Setup.
5.4 Set Job Type: Optimization; Method: Ground State, DFT; Spin: default; Functional: B3LYP; Basis set: 6-31G.
5.5 Set Solvation: None for gas phase; CPCM with water for solvated phase.
5.6 Run calculation.
5.7 Extract HOMO, LUMO, and energy gap from summary and log files.
5.8 Visualize surfaces: Results → Surface/Contours → Cubes (New Orbital) → HOMO/LUMO → New Surface → ON view window → Right click → Display format → Surface → Transparent.
5.9 Generate total density map and mapped surface.

**6. Molecular Dynamics (MD) Simulation**
6.1 Prepare protein topology: gmx pdb2gmx -f REC.pdb -ignh (CHARMM27 = 8, TIP3P = 1).
6.2 Prepare ligand topology: add hydrogens, save as MOL2, upload to SwissParam to generate .itp file.
6.3 Convert ligand to GRO: gmx editconf -f LIG.pdb -o LIG.gro.
6.4 Edit conf.gro, include ligand topology in topol.top as #include "LIG.itp" and add LIG 1.
6.5 Define simulation box: gmx editconf -f conf.gro -d 1.0 -bt triclinic -o box.gro.
6.6 Solvate: gmx solvate -cp box.gro -cs spc216.gro -p topol.top -o box_sol.gro.
6.7 Prepare ions: gmx grompp -f ions.mdp -c box_sol.gro -p topol.top -o ION.tpr; gmx genion -s ION.tpr -p topol.top -conc 0.1 -neutral -o box_sol_ion.gro.
6.8 Energy minimization: gmx grompp -f EM.mdp -c box_sol_ion.gro -maxwarn 2 -p topol.top -o EM.tpr; gmx mdrun -v -deffnm EM.
6.9 Generate ligand position restraints: gmx make_ndx -f LIG.gro -o index_LIG.ndx (> 0 & ! a H* → q); gmx genrestr -f LIG.gro -n index_LIG.ndx -o posre_LIG.itp -fc 1000 1000 1000 (select group 3).
6.10 Add position restraints to protein topology: #ifdef POSRES #include "posre_LIG.itp" #endif.
6.11 Create index groups: gmx make_ndx -f EM.gro -o index.ndx (> 1 | 13 → q).
6.12 NVT equilibration: gmx grompp -f NVT.mdp -c EM.gro -r EM.gro -p topol.top -n index.ndx -maxwarn 2 -o NVT.tpr; gmx mdrun -deffnm NVT.
6.13 NPT equilibration: gmx grompp -f NPT.mdp -c NVT.gro -r NVT.gro -p topol.top -n index.ndx -maxwarn 2 -o NPT.tpr; gmx mdrun -deffnm NPT.
6.14 Production MD: gmx grompp -f MD.mdp -c NPT.gro -t NPT.cpt -p topol.top -n index.ndx -maxwarn 2 -o MD.tpr; gmx mdrun -deffnm MD.
6.15 Center and fit trajectories: gmx trjconv -s MD.tpr -f MD.xtc -o MD_center.xtc -center -pbc mol -ur compact; gmx trjconv -s MD.tpr -f MD_center.xtc -o MD_fit.xtc -fit rot+trans.
6.16 Calculate RMSD, RMSF, Rg, SASA, and H-bonds: gmx rms -s MD.tpr -f MD_center.xtc -o rmsd.xvg -tu ns; gmx rmsf -s MD.tpr -f MD_center.xtc -o rmsf.xvg -res; gmx gyrate -s MD.tpr -f MD_center.xtc -o gyrate1.xvg -tu ns; gmx sasa -f MD_center.xtc -s MD.tpr -o SASA.xvg -tu ns; gmx hbond -s MD.tpr -f MD_center.xtc -num hb.xvg -tu ns.

**7. MM/GBSA Analysis**
Activate conda environment and run:

source ~/miniconda3/etc/profile.d/conda.sh

conda activate gmxMMPBSA

cd /home/Test

gmx_MMPBSA --create_input gb

gmx_MMPBSA -O -i '/home/Test/mmpbsa.in' -cs '/home/Test/MD.tpr' -ct '/home/Test/MD_fit.xtc' -ci '/home/Test/index.ndx' -cg 1 13 -cp '/home/Test/topol.top' -o '/home/Test/Results_MMPBSA.dat' -eo '/home/Test/Results_MMPBSA.csv'

**8. Principal Component Analysis (PCA) and DCCM**
Perform PCA:

echo 21 21 | gmx covar -f MD_fit.xtc -s MD.tpr -n index.ndx -o eigenval.xvg -v eigenvec.trr -tu ns

echo 21 21 | gmx anaeig -v eigenvec.trr -f MD_fit.xtc -eig eigenval.xvg -s MD.tpr -first 1 -last 2 -2d proj.xvg -n index.ndx

DCCM analysis was performed using Python libraries: MDAnalysis, NumPy, Matplotlib, and Seaborn.
