# Supplementary material for: Identification of two terpenoids from Withania coagulans with predicted multitarget binding affinity: An in vitro and in silico study
Source: PLoS One. 2026 Feb 20;21(2):e0343273. doi: 10.1371/journal.pone.0343273 (PMC12923132; doi:10.1371/journal.pone.0343273)
Supplement: S1 Table — (DOCX) [file pone.0343273.s002.docx]

Table S1. Phytochemical constituents identified in the aqueous stem extract of Withania coagulans by GC–MS analysis, along with their PubChem CID, molecular formula, molecular weight, canonical SMILES, and chemical structures.

| **S No.** | **Compound Name** | **PUBCHEM CID** | **Molecular formula** | **Molecular weight (g/mol)** | **Canonical Smiles** | **Structures** |
| --- | --- | --- | --- | --- | --- | --- |
| 1 | p-Fluoroethylbenzene | 136303 | C8H9F | 124.15 | CCC1=CC=C(C=C1)F | 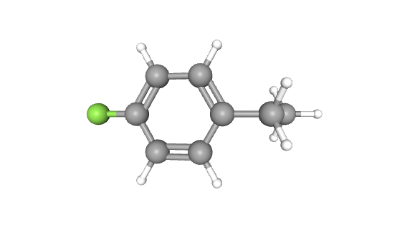 |
| 2 | Cyclohexane, (1-methylethylidene)- | 138578 | C9H16 | 124.22 | CC(=C1CCCCC1)C | 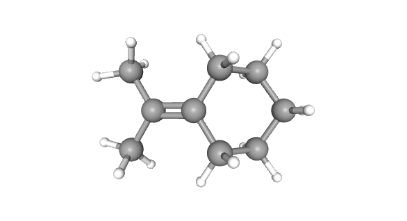 |
| 3 | Cyclopentene, 3-methyl-1-(1-methylethyl)- | 21572743 | C9H16 | 124.22 | CC1CCC(=C1)C(C)C | 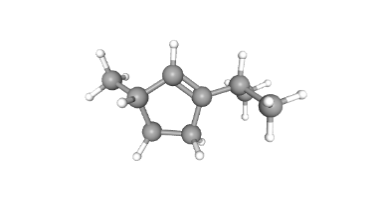 |
| 4 | Benzoic acid | 243 | C7H6O2 | 122.12 | C1=CC=C(C=C1)C(=O)O | 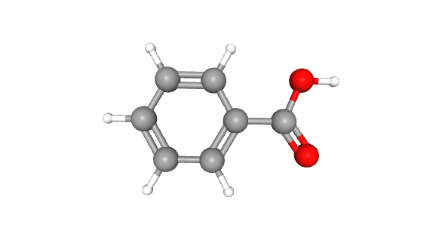 |
| 5 | 2-Methoxy-4-vinylphenol | 332 | C9H10O2 | 150.17 | COC1=C(C=CC(=C1)C=C)O | 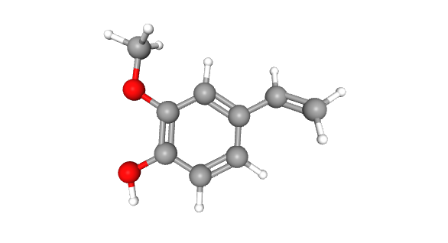 |
| 6 | Dimefox | 8264 | C4H12FN2OP | 154.12 | CN(C)P(=O)(N(C)C)F | 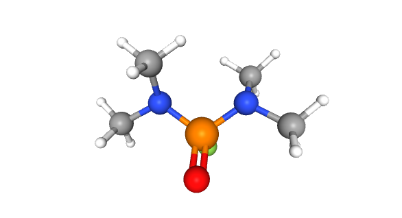 |
| 7 | 2-Butenal, (1-methylethyl)hydrazone | 9602606 | C7H14N2 | 126.20 | C/C=C/C=N/NC(C)C | 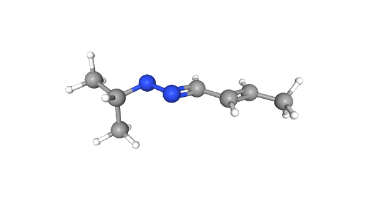 |
| 8 | Carane, 4,5-epoxy-, trans | 119627 | C10H16O | 152.23 | CC1CC2C(C2(C)C)C3C1O3 | 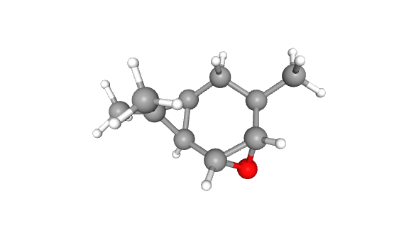 |
| 9 | 2-Cyclohexene-1-carboxaldehyde, trimethyl | 94143 | C10H16O | 152.23 | CC1=CCCC(C1C=O)(C)C | 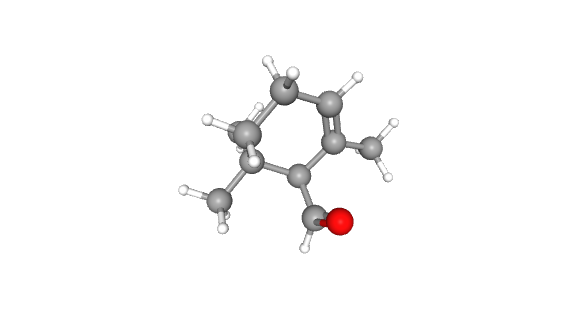 |
| 10 | 7-Thiabicyclo[4.2.1]nonane | 559235 | C8H14S | 142.26 | C1CCC2CC(C1)CS2 | 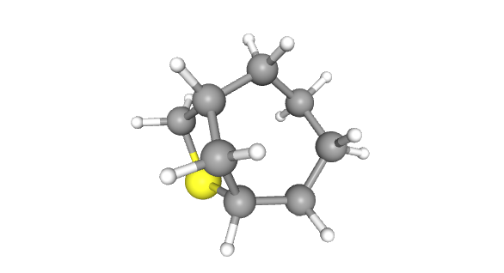 |
| 11 | 2H-Pyran, 2-[(1-butyl-2-propynyl)oxy] | 558910 | C12H20O2 | 196.29 | CCCCC(C#C)OC1CCCCO1 | 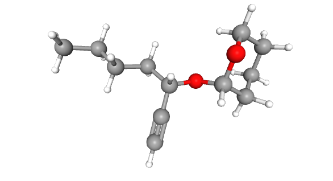 |
| 12 | 2H-Pyran, 2-(7-dodecynyloxy)tetrahydro- | 86051 | C17H30O2 | 266.4 | CCCCC#CCCCCCCOC1CCCCO1 | 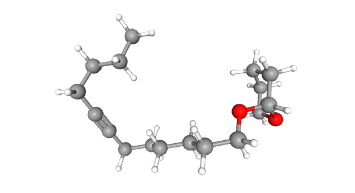 |
| 13 | cis-2,6-Dimethyl-2,6-octadiene | 5352478 | C10H18 | 138.25 | C/C=C(/C)\CCC=C(C)C | 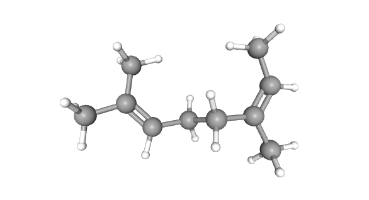 |
| 14 | Nonane, 2-methyl-3-methylene- | 549980 | C11H22 | 154.29 | CCCCCCC(=C)C(C)C | 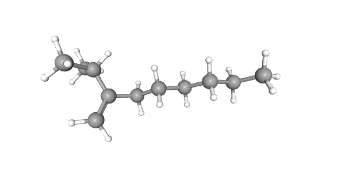 |
| 15 | 3-Cyclohexen-1-carboxaldehyde, 3,4-dimethyl- | 537551 | C9H14O | 138.21 | CC1=C(CC(CC1)C=O)C | 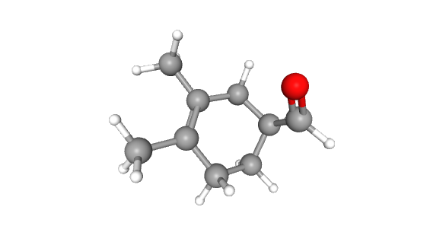 |
| 16 | Propylamine, 3-(furan-2-yl)-1-methyl- | 2772029 | C8H13NO | 139.19 | CC(CCC1=CC=CO1)N | 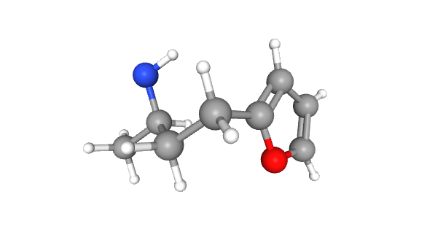 |
| 17 | Beta-Myrcene | 31253 | C10H16 | 136.23 | CC(=CCCC(=C)C=C)C | 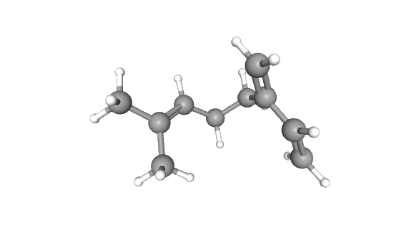 |
| 18 | Hexadecanoic acid, methyl ester | 8181 | C17H34O2 | 270.5 | CCCCCCCCCCCCCCCC(=O)OC | 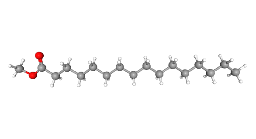 |
| 19 | n-Hexadecanoic acid | 985 | C16H32O2 | 256.42 | CCCCCCCCCCCCCCCC(=O)O | 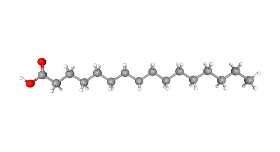 |
| 20 | Methyl 8-methyl-nonanoate | 20619411 | C11H22O2 | 186.29 | CC(C)CCCCCCC(=O)OC | 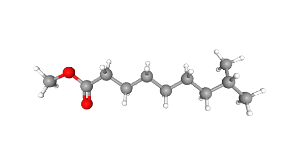 |
| 21 | Cyclopropane carboxamide, 2-cyclopropyl-2-methyl-N-(1-cyclopropylethyl)- | 534988 | C13H21NO | 207.31 | CC(C1CC1)NC(=O)C2CC2(C)C3CC3 | 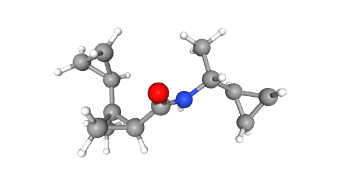 |
| 22 | Dimethyl N,N-dimethylphosphoramidate | 69003 | C4H12NO3P | 153.12 | CN(C)P(=O)(OC)OC | 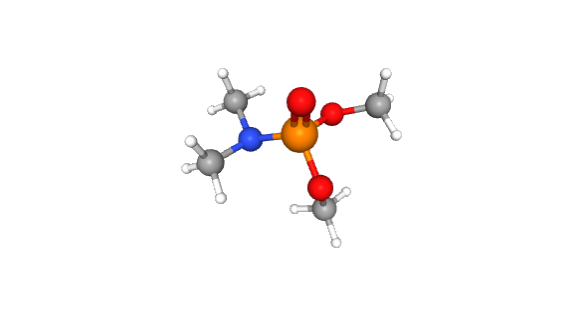 |
| 23 | 1-Methylene-2b-hydroxymethyl-3,3-dimethyl-4b-(3-methylbut-2-enyl)-cyclohexane | 550196 | C15H26O | 222.37 | CC(=CCC1CC(=C)C(C(C1)(C)C)CO)C | 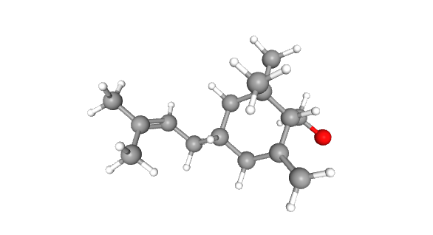 |
| 24 | 3-Methoxybenzyl alcohol | 81437 | C8H10O2 | 138.16 | COC1=CC=CC(=C1)CO | 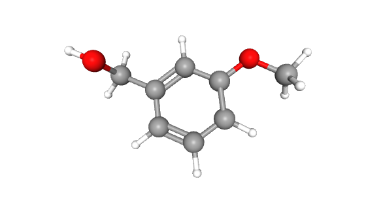 |
| 25 | Caryophyllene oxide | 1742210 | C15H24O | 220.35 | C[C@@]12CC[C@@H]3[C@H](CC3(C)C)C(=C)CC[C@H]1O2 | 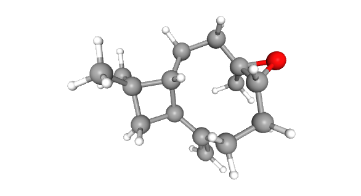 |
| 26 | N-Methyl-3-(methylamino)propanamide | 12548977 | C5H12N2O | 116.16 | CNCCC(=O)NC | 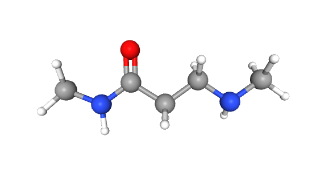 |
| 27 | 4,4-Dimethyl-cyclohex-2-en-1-ol | 543508 | C8H14O | 126.20 | CC1(CCC(C=C1)O)C | 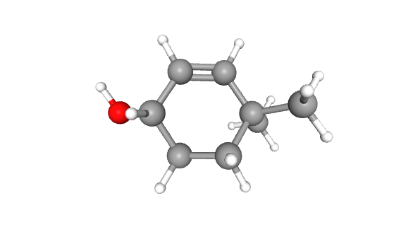 |
| 28 | 2,2-Dimethyl-3-(3,7,16,20-tetramethylheneicosa-3,7,11,15,19-pentaenyl)-oxirane | 693 | C30H50O | 426.7 | CC(=CCCC(=CCCC(=CCCC=C(C)CCC=C(C)CCC1C(O1)(C)C)C)C)C | 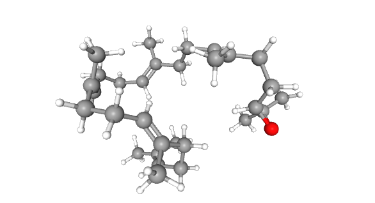 |
| 29 | 4-Hydroxy-3-methylacetophenone | 70135 | C9H10O2 | 150.17 | CC1=C(C=CC(=C1)C(=O)C)O | 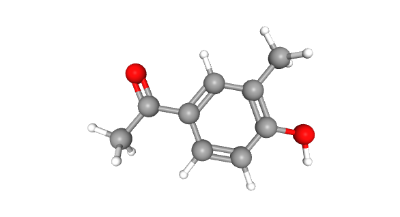 |
| 30 | Methyl ethyl cyclopentene | 88243 | C8H14 | 110.20 | CCC1=C(CCC1)C | 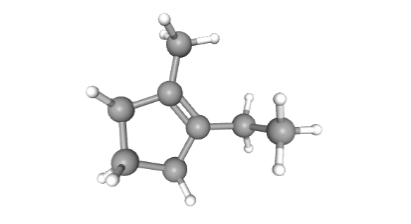 |
| 31 | 1-H-Indene, octahydro-, trans | 638055 | C9H16 | 124.22 | C1CC[C@@H]2CCC[C@H]2C1 | 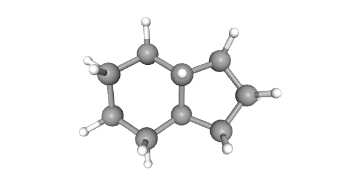 |
| 32 | Cyclohexane, 1,1,2,3-tetramethyl- | 549623 | C10H20 | 140.27 | CC1CCCC(C1C)(C)C | 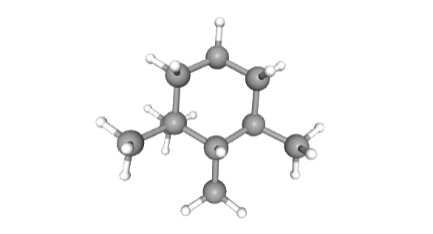 |
