# Supplementary material for: Identification of two terpenoids from Withania coagulans with predicted multitarget binding affinity: An in vitro and in silico study
Source: PLoS One. 2026 Feb 20;21(2):e0343273. doi: 10.1371/journal.pone.0343273 (PMC12923132; doi:10.1371/journal.pone.0343273)
Supplement: S2 Table — (DOCX) [file pone.0343273.s003.docx]

Table S2. Drug-likeness and pharmacokinetic properties of GCMS identified compounds.

| **Compounds** | **TPSA** | **GI absorption** | **BBB permeant** | **CYP2D6 inhibitor** | **CYP3A4 inhibitor** | **CYP1A2 inhibitor** | **Lipinski** | **Veber** | **Egan** | **Log Kp (skin permeation)** | **Bioavailability Score** |
| --- | --- | --- | --- | --- | --- | --- | --- | --- | --- | --- | --- |
| p-Fluoroethylbenzene | 0.00 Å² | Low | Yes | No | No | Yes | 0 | 0 | 0 | -5.11 | 0.55 |
| Cyclohexane, (1-methylethylidene)- | 0.00 Å² | Low | Yes | No | No | No | 0 | 0 | 0 | -4.59 | 0.55 |
| Cyclopentene, 3-methyl-1-(1-methylethyl)- | 0.00 Å² | Low | Yes | No | No | No | 0 | 0 | 0 | -4.98 | 0.55 |
| Benzoic acid | 37.30 Å² | High | Yes | No | No | No | 0 | 0 | 0 | -5.72 | 0.85 |
| 2-Methoxy-4-vinylphenol | 29.46 Å² | High | Yes | No | No | Yes | 0 | 0 | 0 | -5.22 | 0.55 |
| Dimefox | 33.36 Å² | High | Yes | No | No | No | 0 | 0 | 0 | -7.33 | 0.55 |
| 2-Butenal, (1-methylethyl)hydrazone | 24.39 Å² | High | Yes | No | No | No | 0 | 0 | 0 | -5.69 | 0.55 |
| Carane, 4,5-epoxy-, trans | 12.53 Å² | High | Yes | No | No | No | 0 | 0 | 0 | -5.53 | 0.55 |
| 2-Cyclohexene-1-carboxaldehyde, trimethyl | 17.07 Å² | High | Yes | No | No | No | 0 | 0 | 0 | -5.67 | 0.55 |
| 7-Thiabicyclo[4.2.1]nonane | 25.30 Å² | High | Yes | No | No | No | 0 | 0 | 0 | -5.24 | 0.55 |
| 2H-Pyran, 2-[(1-butyl-2-propynyl)oxy] | 18.46 Å² | High | Yes | No | No | No | 0 | 0 | 0 | -5.45 | 0.55 |
| 2H-Pyran, 2-(7-dodecynyloxy)tetrahydro- | 18.46 Å² | High | Yes | Yes | No | Yes | 0 | 0 | 0 | -4.13 | 0.55 |
| cis-2,6-Dimethyl-2,6-octadiene | 0.00 Å² | Low | Yes | No | No | No | 0 | 0 | 0 | -4.2 | 0.55 |
| Nonane, 2-methyl-3-methylene- | 0.00 Å² | Low | Yes | No | No | No | 1 | 0 | 0 | -3.39 | 0.55 |
| 3-Cyclohexen-1-carboxaldehyde, 3,4-dimethyl- | 17.07 Å² | High | Yes | No | No | No | 0 | 0 | 0 | -6.4 | 0.55 |
| Propylamine, 3-(furan-2-yl)-1-methyl- | 39.16 Å² | High | Yes | No | No | Yes | 0 | 0 | 0 | -6.06 | 0.55 |
| Beta-Myrcene | 0.00 Å² | Low | Yes | No | No | No | 0 | 0 | 0 | -4.17 | 0.55 |
| Hexadecanoic acid, methyl ester | 26.30 Å² | High | Yes | No | No | Yes | 1 | 1 | 0 | -2.71 | 0.55 |
| n-Hexadecanoic acid | 37.30 Å² | High | Yes | No | No | Yes | 1 | 1 | 0 | -2.77 | 0.85 |
| Methyl 8-methyl-nonanoate | 26.30 Å² | High | Yes | No | No | No | 0 | 0 | 0 | -4.19 | 0.55 |
| Cyclopropane carboxamide, 2-cyclopropyl-2-methyl-N-(1-cyclopropylethyl)- | 29.10 Å² | High | Yes | No | No | No | 0 | 0 | 0 | -5.73 | 0.55 |
| Dimethyl N,N-dimethylphosphoramidate | 48.58 Å² | High | Yes | No | No | No | 0 | 0 | 0 | -7.44 | 0.55 |
| 1-Methylene-2b-hydroxymethyl-3,3-dimethyl-4b-(3-methylbut-2-enyl)-cyclohexane | 20.23 Å² | High | Yes | No | No | No | 0 | 0 | 0 | -4.78 | 0.55 |
| 3-Methoxybenzyl alcohol | 29.46 Å² | High | Yes | No | No | No | 0 | 0 | 0 | -9.71 | 0.55 |
| Caryophyllene oxide | 12.53 Å² | High | Yes | No | No | No | 0 | 0 | 0 | -5.12 | 0.55 |
| N-Methyl-3-(methylamino)propanamide | 41.13 Å² | High | No | No | No | No | 0 | 0 | 0 | -7.68 | 0.55 |
| 4,4-Dimethyl-cyclohex-2-en-1-ol | 20.23 Å² | High | Yes | No | No | No | 0 | 0 | 0 | -5.75 | 0.55 |
| 2,2-Dimethyl-3-(3,7,16,20-tetramethylheneicosa-3,7,11,15,19-pentaenyl)-oxirane | 12.53 Å² | Low | No | No | No | Yes | 1 | 1 | 1 | -1.6 | 0.55 |
| 4-Hydroxy-3-methylacetophenone | 37.30 Å² | High | Yes | No | No | Yes | 0 | 0 | 0 | -6.54 | 0.55 |
| Methyl ethyl cyclopentene | 0.00 Å² | Low | Yes | No | No | No | 0 | 0 | 0 | -5.42 | 0.55 |
| 1-H-Indene, octahydro-, trans | 0.00 Å² | Low | Yes | No | No | No | 0 | 0 | 0 | -4.16 | 0.55 |
| Cyclohexane, 1,1,2,3-tetramethyl- | 0.00 Å² | Low | Yes | No | No | No | 1 | 0 | 0 | -4.03 | 0.55 |
