# Supplementary material for: Identification of two terpenoids from Withania coagulans with predicted multitarget binding affinity: An in vitro and in silico study
Source: PLoS One. 2026 Feb 20;21(2):e0343273. doi: 10.1371/journal.pone.0343273 (PMC12923132; doi:10.1371/journal.pone.0343273)
Supplement: S3 Table — (DOCX) [file pone.0343273.s004.docx]

Table S3. Cross Validation of ADME properties of selected compounds by SwissADME and pkCSM.

| Molecule | Caryophyllene oxide | | | |
| --- | --- | --- | --- | --- |
| Model name | Unit (pKCSM) | pKCSM Prediction | Unit (SwissADME) | SwissADME Prediction |
| Water solubility (consensus) | log mol/L | –4.5 | mol/L | 3.56E-04 |
| GI absorption (human) | % absorbed | 98.609 | High/Low | High |
| Skin Permeability | log Kp | –2.96 | log Kp | –5.12 |
| P-glycoprotein substrate | Yes/No | Yes | Yes/No | No |
| P-glycoprotein inhibitor | Yes/No | No | — | — |
| VDss (human) | log L/kg | 0.733 | — | — |
| Fraction unbound (human) | Fu | 0.319 | — | — |
| BBB permeability | log BB | 0.518 | Yes/No | Yes |
| CNS permeability | log PS | –2.157 | — | — |
| CYP2D6 substrate | Yes/No | No | Yes/No | No |
| CYP3A4 substrate | Yes/No | No | Yes/No | No |
| CYP1A2 inhibitor | Yes/No | Yes | Yes/No | No |
| CYP2C19 inhibitor | Yes/No | No | Yes/No | No |
| CYP2C9 inhibitor | Yes/No | No | Yes/No | No |
| CYP2D6 inhibitor | Yes/No | No | Yes/No | No |
| CYP3A4 inhibitor | Yes/No | No | Yes/No | No |
| Total Clearance | log mL/min/kg | 0.705 | — | — |
| Renal OCT2 substrate | Yes/No | No | — | — |

| Molecule | 2,2-Dimethyl-3-(3,7,16,20-tetramethylheneicosa-3,7,11,15,19-pentaenyl)-oxirane | | | |
| --- | --- | --- | --- | --- |
| Model name | **Unit (pKCSM)** | **pKCSM Prediction** | **Unit (SwissADME)** | **SwissADME Prediction** |
| Water solubility (consensus) | log mol/L | –8.164 | mol/L | 1.07E-08 |
| GI absorption (human) | % absorbed | 92.219 | High/Low | Low |
| Skin Permeability | log Kp | –3.253 | log Kp | –1.6 |
| P-glycoprotein substrate | Yes/No | Yes | Yes/No | Yes |
| P-glycoprotein inhibitor | Yes/No | Yes (I & II) | — | — |
| VDss (human) | log L/kg | 1.161 | — | — |
| Fraction unbound (human) | Fu | 0.0 | — | — |
| BBB permeability | log BB | 1.045 | Yes/No | No |
| CNS permeability | log PS | –0.803 | — | — |
| CYP2D6 substrate | Yes/No | No | Yes/No | No |
| CYP3A4 substrate | Yes/No | Yes | Yes/No | No |
| CYP1A2 inhibitor | Yes/No | No | Yes/No | No |
| CYP2C19 inhibitor | Yes/No | No | Yes/No | No |
| CYP2C9 inhibitor | Yes/No | No | Yes/No | No |
| CYP2D6 inhibitor | Yes/No | No | Yes/No | No |
| CYP3A4 inhibitor | Yes/No | No | Yes/No | No |
| Total Clearance | log mL/min/kg | 1.359 | — | — |
| Renal OCT2 substrate | Yes/No | No | — | — |
