# Supplementary material for: Identification of two terpenoids from Withania coagulans with predicted multitarget binding affinity: An in vitro and in silico study
Source: PLoS One. 2026 Feb 20;21(2):e0343273. doi: 10.1371/journal.pone.0343273 (PMC12923132; doi:10.1371/journal.pone.0343273)
Supplement: S4 Table — (DOCX) [file pone.0343273.s005.docx]

Table S4: Docking Score of GCMS identified Compounds with targeted Proteins.

| **S No.** | **Compound Name** | **AMY2A**  Homo sapiens  **PDB ID: 5U3A** | **HipBST**  Escherichia coli  **PDB ID: 7AB4** | **KEAP1**  Homo sapiens  **PDB ID: 7Q6S** | **ompF**  Salmonella Typhi  **PDB ID: 4KR4** |
| --- | --- | --- | --- | --- | --- |
| 1 | p-Fluoroethylbenzene | -5.1 | -5.0 | -5.2 | -4.3 |
| 2 | Cyclohexane, (1-methylethylidene)- | -5.1 | -4.9 | -5.3 | -4.4 |
| 3 | Cyclopentene, 3-methyl-1-(1-methylethyl)- | -5.1 | -5.1 | -5.4 | -4.7 |
| 4 | Benzoic acid | -5.4 | -5.7 | -5.6 | -4.7 |
| 5 | 2-Methoxy-4-vinylphenol | -5.6 | -5.1 | -5.9 | -4.6 |
| 6 | Dimefox | -3.7 | -3.7 | -4.1 | -3.7 |
| 7 | 2-Butenal, (1-methylethyl)hydrazone | -4.8 | -4.9 | -4.6 | -4.3 |
| 8 | Carane, 4,5-epoxy-, trans | -5.8 | -5.3 | -5.3 | -4.7 |
| 9 | 2-Cyclohexene-1-carboxaldehyde, trimethyl | -5.3 | -5.1 | -5.4 | -4.6 |
| 10 | 7-Thiabicyclo[4.2.1]nonane | -4.7 | -4.3 | -4.7 | -4.0 |
| 11 | 2H-Pyran, 2-[(1-butyl-2-propynyl)oxy] | -5.5 | -5.2 | -5.9 | -4.8 |
| 12 | 2H-Pyran, 2-(7-dodecynyloxy)tetrahydro- | -5.5 | -5.6 | -6.0 | -4.6 |
| 13 | cis-2,6-Dimethyl-2,6-octadiene | -5.3 | -4.9 | -5.1 | -4.2 |
| 14 | Nonane, 2-methyl-3-methylene- | -4.8 | -4.1 | -4.8 | -3.7 |
| 15 | 3-Cyclohexen-1-carboxaldehyde, 3,4-dimethyl- | -5.2 | -5.1 | -5.2 | -5.0 |
| 16 | Propylamine, 3-(furan-2-yl)-1-methyl- | -4.7 | -4.8 | -4.7 | -4.4 |
| 17 | Beta-Myrcene | -4.8 | -4.5 | -4.9 | -4.0 |
| 18 | Hexadecanoic acid, methyl ester | -4.6 | -4.5 | -5.5 | -4.2 |
| 19 | n-Hexadecanoic acid | -4.8 | -5.0 | -5.4 | -4.3 |
| 20 | Methyl 8-methyl-nonanoate | -5.0 | -4.3 | -5.2 | -4.0 |
| 21 | Cyclopropane carboxamide, 2-cyclopropyl-2-methyl-N-(1-cyclopropylethyl)- | -5.7 | -5.5 | -5.6 | -4.9 |
| 22 | Dimethyl N,N-dimethylphosphoramidate | -3.5 | -4.0 | -4.0 | -3.6 |
| 23 | 1-Methylene-2b-hydroxymethyl-3,3-dimethyl-4b-(3-methylbut-2-enyl)-cyclohexane | -6.8 | -5.9 | -6.3 | -5.5 |
| 24 | 3-Methoxybenzyl alcohol | -5.1 | -4.8 | -5.2 | -4.4 |
| 25 | Caryophyllene oxide | -7.2 | -7.4 | -6.3 | -5.6 |
| 26 | N-Methyl-3-(methylamino)propanamide | -3.6 | -4.0 | -3.7 | -3.4 |
| 27 | 4,4-Dimethyl-cyclohex-2-en-1-ol | -5.0 | -4.7 | -4.9 | -4.5 |
| 28 | 2,2-Dimethyl-3-(3,7,16,20-tetramethylheneicosa-3,7,11,15,19-pentaenyl)-oxirane | -6.9 | -7.6 | -7.5 | -6.3 |
| 29 | 4-Hydroxy-3-methylacetophenone | -5.7 | -5.5 | -6.1 | -5.2 |
| 30 | Methyl ethyl cyclopentene | -4.8 | -4.2 | -4.7 | -4.4 |
| 31 | 1-H-Indene, octahydro-, trans | -5.4 | -5.5 | -5.3 | -4.5 |
| 32 | Cyclohexane, 1,1,2,3-tetramethyl- | -5.8 | -5.0 | -5.2 | -4.7 |
