# Supplementary material for: Identification of two terpenoids from Withania coagulans with predicted multitarget binding affinity: An in vitro and in silico study
Source: PLoS One. 2026 Feb 20;21(2):e0343273. doi: 10.1371/journal.pone.0343273 (PMC12923132; doi:10.1371/journal.pone.0343273)
Supplement: Attachment — Submitted filename: Point_by_point_response_plos_One Final.docx [file pone.0343273.s008.docx]

**AHMED VANDY**

[ahmedvandy@gmail.com](mailto:ahmedvandy@gmail.com) / +232-79-522721

[LinkedIn](https://www.linkedin.com/in/ahmed-vandy-964201159/) / [ORCID](https://orcid.org/0009-0008-2603-9989)

14^th^ December, 2025.

Dear Reviewers and Editorial Team,

We sincerely thank the Academic Editor and all the reviewers for their time, valuable feedback, and insightful comments on our manuscript. We appreciate the opportunity to revise our work and address the critical points that were raised.

The review process has significantly helped us improve the scientific quality, clarity, and presentation of our study. We have carefully revised the manuscript and responded to each comment point-by-point. We are grateful for your guidance and constructive suggestions, which contributed to refining the methodology, strengthening the interpretation of results, and improving overall transparency and reproducibility.

We hope the revised version meets the journal’s standards and expectations, and we look forward to the possibility of publication in PLOS ONE.

PONE-D-25-53146

Identification of Two Terpenoids from Withania coagulans as Multi-Target Inhibitors: An In Vitro and In Silico Study

PLOS ONE

Dear Dr. Vandy,

Thank you for submitting your manuscript to PLOS ONE. After careful consideration, we feel that it has merit but does not fully meet PLOS ONE’s publication criteria as it currently stands. Therefore, we invite you to submit a revised version of the manuscript that addresses the points raised during the review process.

==============================

The submission reflects scientific relevance. However, some fundamental issues limit its quality for publication in the current form. For instance, the authors need to justify the significance of the study, relate it to the literature and identify the gap in the existing knowledge that this study aims to address, and ensure an adequate validation of the theoretical analyses reported. Again, what are the limitations of this study, and how can the authors recommend future research on the study? Moreover, some serious concerns have been raised by the reviewers affecting pivotal sections of the study. Kindly pay close attention to these and address them critically before resubmission.

==============================

Please submit your revised manuscript by Dec 06 2025 11:59PM. If you will need more time than this to complete your revisions, please reply to this message or contact the journal office at plosone@plos.org. When you're ready to submit your revision, log on to https://www.editorialmanager.com/pone/ and select the 'Submissions Needing Revision' folder to locate your manuscript file.

Please include the following items when submitting your revised manuscript:

A rebuttal letter that responds to each point raised by the academic editor and reviewer(s). You should upload this letter as a separate file labeled 'Response to Reviewers'.

A marked-up copy of your manuscript that highlights changes made to the original version. You should upload this as a separate file labeled 'Revised Manuscript with Track Changes'.

An unmarked version of your revised paper without tracked changes. You should upload this as a separate file labeled 'Manuscript'.

If you would like to make changes to your financial disclosure, please include your updated statement in your cover letter. Guidelines for resubmitting your figure files are available below the reviewer comments at the end of this letter.

If applicable, we recommend that you deposit your laboratory protocols in protocols.io to enhance the reproducibility of your results. Protocols.io assigns your protocol its own identifier (DOI) so that it can be cited independently in the future. For instructions see: https://journals.plos.org/plosone/s/submission-guidelines#loc-laboratory-protocols. Additionally, PLOS ONE offers an option for publishing peer-reviewed Lab Protocol articles, which describe protocols hosted on protocols.io. Read more information on sharing protocols at https://plos.org/protocols?utm_medium=editorial-email&utm_source=authorletters&utm_campaign=protocols.

We look forward to receiving your revised manuscript.

Kind regards,

Yusuf Oloruntoyin Ayipo, Ph.D

Academic Editor

PLOS ONE

Journal Requirements:

1. When submitting your revision, we need you to address these additional requirements.

Please ensure that your manuscript meets PLOS ONE's style requirements, including those for file naming. The PLOS ONE style templates can be found at

https://journals.plos.org/plosone/s/file?id=wjVg/PLOSOne_formatting_sample_main_body.pdf and

<https://journals.plos.org/plosone/s/file?id=ba62/PLOSOne_formatting_sample_title_authors_affiliations.pdf>

**Authors response:** Thank you for the comment. We have reshaped the manuscript formatting to meets PLOS ONE's style requirements. Font size and Figure labeling has been revised.

2. Please include a complete copy of PLOS’ questionnaire on inclusivity in global research in your revised manuscript. Our policy for research in this area aims to improve transparency in the reporting of research performed outside of researchers’ own country or community. The policy applies to researchers who have travelled to a different country to conduct research, research with Indigenous populations or their lands, and research on cultural artefacts. The questionnaire can also be requested at the journal’s discretion for any other submissions, even if these conditions are not met. Please find more information on the policy and a link to download a blank copy of the questionnaire here: https://journals.plos.org/plosone/s/best-practices-in-research-reporting. Please upload a completed version of your questionnaire as Supporting Information when you resubmit your manuscript.

**Authors response:** The form has been completed and uploaded together with the submission.

3. In your Methods section, please provide additional information regarding the permits you obtained for the work. Please ensure you have included the full name of the authority that approved the field site access and, if no permits were required, a brief statement explaining why.

**Authors response:** This study did not involve human participants, indigenous communities, or the collection of materials requiring access or benefit-sharing agreements. Plant stems of Withania coagulans (Dunal) were collected from the Cholistan Desert, Punjab, Pakistan, on privately owned land belonging to the authors, where the species grows naturally. Botanical authentication was conducted by Muhammad Numan Khan, Department of Botany, Islamia College Peshawar. As the collection occurred on private property and did not involve protected or endangered species, no permits or licenses were required.

4. Please note that PLOS One has specific guidelines on code sharing for submissions in which author-generated code underpins the findings in the manuscript. In these cases, we expect all author-generated code to be made available without restrictions upon publication of the work. Please review our guidelines at https://journals.plos.org/plosone/s/materials-and-software-sharing#loc-sharing-code and ensure that your code is shared in a way that follows best practice and facilitates reproducibility and reuse.

**Authors response:** The raw data has been uploaded with the submission.

5. Please upload a new copy of Figures 6, 8, 10 and 11, as the detail is not clear. Please follow the link for more information: https://journals.plos.org/plosone/s/figures

**Authors response:** Done as requested, Thank you.

6. Please include captions for your Supporting Information files at the end of your manuscript, and update any in-text citations to match accordingly. Please see our Supporting Information guidelines for more information: <http://journals.plos.org/plosone/s/supporting-information>.

**Authors response:** Done as requested, Thank you.

7. If the reviewer comments include a recommendation to cite specific previously published works, please review and evaluate these publications to determine whether they are relevant and should be cited. There is no requirement to cite these works unless the editor has indicated otherwise.

**Authors response:** Thank you for the instruction

Additional Editor Comments:

The submission reflects scientific relevance. However, some fundamental issues limit its quality for publication in the current form. For instance, the authors need to justify the significance of the study, relate it to the literature and identify the gap in the existing knowledge that this study aims to address, and ensure an adequate validation of the theoretical analyses reported. Again, what are the limitations of this study, and how can the authors recommend future research on the study? Moreover, some serious concerns have been raised by the reviewers affecting pivotal sections of the study. Kindly pay close attention to these and address them critically before resubmission.

**Authors response:** Thank you for the comments, the manuscript has now been revised.

**Authors response:**

[Note: HTML markup is below. Please do not edit.]

Reviewers' comments:

Reviewer's Responses to Questions

Comments to the Author

1. Is the manuscript technically sound, and do the data support the conclusions?

The manuscript must describe a technically sound piece of scientific research with data that supports the conclusions. Experiments must have been conducted rigorously, with appropriate controls, replication, and sample sizes. The conclusions must be drawn appropriately based on the data presented.

Reviewer #1: Yes

Reviewer #2: Yes

Reviewer #3: Partly

Reviewer #4: Yes

Reviewer #5: Yes

2. Has the statistical analysis been performed appropriately and rigorously?

Reviewer #1: No

Reviewer #2: N/A

Reviewer #3: Yes

Reviewer #4: Yes

Reviewer #5: No

3. Have the authors made all data underlying the findings in their manuscript fully available?

The PLOS Data policy requires authors to make all data underlying the findings described in their manuscript fully available without restriction, with rare exception (please refer to the Data Availability Statement in the manuscript PDF file). The data should be provided as part of the manuscript or its supporting information, or deposited to a public repository. For example, in addition to summary statistics, the data points behind means, medians and variance measures should be available. If there are restrictions on publicly sharing data—e.g. participant privacy or use of data from a third party—those must be specified.

Reviewer #1: Yes

Reviewer #2: No

Reviewer #3: Yes

Reviewer #4: Yes

Reviewer #5: No

4. Is the manuscript presented in an intelligible fashion and written in standard English?

PLOS ONE does not copyedit accepted manuscripts, so the language in submitted articles must be clear, correct, and unambiguous. Any typographical or grammatical errors should be corrected at revision, so please note any specific errors here.

Reviewer #1: Yes

Reviewer #2: Yes

Reviewer #3: Yes

Reviewer #4: Yes

Reviewer #5: Yes

5. Review Comments to the Author

Please use the space provided to explain your answers to the questions above. You may also include additional comments for the author, including concerns about dual publication, research ethics, or publication ethics. (Please upload your review as an attachment if it exceeds 20,000 characters)

**Reviewer #1**

Reviewer #1: The manuscript provides a comprehensive and original study of Withania coagulans, integrating phytochemical profiling with computational validation. Although the computational study is well described, the microbiological analysis lacks information. Please, see my comments below:

1. Introduction

Consider highlighting in the introduction how this study advances beyond previous work on W. coagulans and terpenoids, to further emphasize its novelty.

**Authors Response:** We thank the reviewer for this insightful suggestion. In response, we have revised the Introduction to more clearly highlight how the present study advances beyond previous work on *Withania coagulans* and its terpenoid constituents. The revised text emphasizes the existing gaps in the literature particularly the limited characterization of terpenoids and the absence of integrated *in vitro*–in silico approaches and explains how our study fills these gaps.

2. Materials and Methods

a) Ensure that all assay conditions (e.g., concentrations, incubation times, controls) are explicitly stated for each experiment.

**Authors response:** We thank the reviewer for this important comment. We have carefully revised the Materials and Methods section to ensure that all assay conditions—including concentrations, volumes, incubation times, temperatures, and control details—are clearly and explicitly described for each experiment.

b) Mention which guidelines (CLSI or EUCAST) are followed to interpret the zone of inhibition. Consider carrying out MIC and MBC assay.

**Authors response:** Thank you for this insightful suggestion. We have updated (Antibacterial Assay) to clearly state that zone of inhibition results were interpreted according to the CLSI M100 (2020) guidelines. Additionally, we have now performed MIC and MBC assays using the broth microdilution method following CLSI M07-A9 protocols, as recommended.

c) The number of replicates for each sample and control (e.g., biological replicates, technical replicates) is not mentioned. This should be clearly stated to ensure statistical relevance.

**Authors response:** We thank the reviewer for this important observation. We have now clarified the number of replicates used in all experiments.

d) The study does not address the potential cytotoxicity of compounds, which is vital for clinical applications.

**Authors response:** We thank the reviewer for this important observation. We acknowledge that assessing cytotoxicity is critical for evaluating the clinical relevance of the identified terpenoids. While the current study focused on phytochemical profiling, in vitro antibacterial, antidiabetic, and antioxidant activity, and in silico analyses, we did not perform experimental cytotoxicity assays.

We have now included a statement in the Discussion and Limitations sections acknowledging this limitation and indicating that future studies will incorporate cytotoxicity testing using appropriate mammalian cell lines to evaluate safety and therapeutic potential. This ensures transparency and aligns with clinical relevance considerations.

e) Consider performing *in-vivo* antidiabetic assay on mice model, and if possible, carryout histopathological test.

**Authors response:** We sincerely thank the reviewer for this valuable suggestion. We fully acknowledge that in vivo antidiabetic evaluation and histopathological analysis would provide important insights into the therapeutic potential and safety of the identified compounds.

However, due to facility limitations and the lack of ethical approval for animal experiments at the current stage, we were unable to perform these assays. We have noted this as a limitation in the manuscript and plan to include in vivo antidiabetic studies and histopathological analysis in future work as part of our ongoing research. We appreciate the reviewer’s understanding and constructive feedback.

3. Results

Compare the results of antibacterial assay with standard in the light of CLSI and/or EUCAST guidelines

**Authors response:** We thank the reviewer for this valuable suggestion. The antibacterial assay results have now been interpreted according to the CLSI (M100) guidelines. Compared with the standard antibiotic (erythromycin), *Withania coagulans* extract exhibited a concentration-dependent inhibitory effect against *Salmonella Typhi* and *Escherichia coli*.

4. Discussions

a) The discussion could be expanded to further explore the broader implications of the findings, including potential applications in drug development and how these compounds compare to existing therapeutics.

**Authors response:** We thank the reviewer for this constructive suggestion. The Discussion section has been expanded to better highlight the broader implications of our findings in the context of drug development.

b) Consider discussing any limitations of the study and possible directions for future research.

Suggestions:

**Authors response:** We thank the reviewer for highlighting this point. We have now included a clear discussion of the study’s limitations and outlined directions for future research

•Revise sections with lengthy or complex sentences to improve clarity and readability.

**Authors response: W**e are grateful for the reviewer's guidance. We have performed a comprehensive revision of the entire manuscript to improve clarity and readability.

•Ensure consistency in terminology throughout the manuscript.

**Authors response:** Thank you for pointing this out. We have carefully revised the manuscript to ensure terminology is consistent throughout.

•Expand figure and table legends to be fully self-explanatory.

**Authors response:** We sincerely thank the reviewer suggestion. We have thoroughly revised all figure and table legends throughout the manuscript.

**Reviewer #2**

Reviewer #2: The integration of phytochemical profiling, biological assays, and computational modeling addresses a relevant topic in medicinal-plant research. The authors attempt to connect in-vitro results with in-silico validation. The selection of Withania coagulans, a well-known ethnomedicinal species, is of genuine pharmacological interest and aligns with the journal’s interdisciplinary scope.

Following are my specific comments for the authors:

The extraction procedure is well described, but the authors should report the extraction yield (% w/w), specify the working extract concentration, and briefly justify the choice of water as the solvent. This information is helpful for reproducibility and comparison with earlier studies.

**Authors response:** We thank the reviewer for this constructive comment. In response, we have updated the Materials and Methods section to include the extraction yield.

The inclusion of at least one Gram-positive strain is suggested (not required though). But the inhibition zones data should be presented with means ± SD from triplicates and statistically compared to the control.

**Authors response:** We sincerely thank the reviewer for this valuable suggestion. Although the inclusion of a Gram-positive strain would further strengthen the antibacterial evaluation, our current study design and available isolates did not allow the addition of new bacterial strains at this stage. However, we acknowledge the importance of assessing both Gram-positive and Gram-negative bacteria, and we will incorporate Gram-positive strains in future studies.

As recommended, the inhibition-zone data have been updated to include mean ± SD values from triplicate experiments, and statistical comparisons with the control have been incorporated in the revised manuscript.

The starch hydrolysis assay based on inhibition zone measurement is unconventional. Please add brief rationale and citation.

**Authors response:** Thank you for your comment. We have now added a brief rationale for using the starch hydrolysis assay based on inhibition zone measurement. This approach is commonly used to assess enzyme activity, specifically for amylase inhibition, as it provides a clear, quantifiable measure of the effect of inhibitors on starch breakdown. We have also included a relevant citation to support this method in the revised manuscript.

The ADMET table gives nearly identical results for all compounds despite their structural diversity? A comment.

**Authors response:** We thank the reviewer for this insightful observation. The similarity in predicted ADMET properties arises because the computational tools used (SwissADME, pkCSM, and ProTox) rely on generalized QSAR models and broad molecular descriptors. Although the compounds differ structurally, many share overlapping physicochemical features—such as comparable lipophilicity, low polarity, and limited hydrogen-bonding capacity—which can produce similar predictive outputs. We have now added a clarifying statement to the manuscript noting that these results represent preliminary in-silico estimations and require future experimental validation.

The paper repeatedly describes the two terpenoids as “multi-target inhibitors” and “potential drug candidates.” One would be more cautious as the study is limited to preliminary in-vitro assays and in-silico predictions.

**Authors response:** Thank you for your thoughtful and constructive feedback. We fully agree with your concern regarding overstatements related to “multi-target inhibitors” and “drug candidates.” To address this, we have carefully revised the entire manuscript to ensure the terminology reflects the preliminary nature of the study.

The discussion also includes statements, for example, assigning cardiovascular or anticancer properties to compounds that were neither tested nor cited from primary literature. Wherever possible, the authors should avoid extrapolations beyond the evidence.

**Authors response:** Thank you for pointing this out. We have now carefully revised the Discussion section, removing or rephrasing speculative statements related to cardiovascular, anticancer, analgesic, or neuroprotective properties of phytoconstituents that were not directly tested in this study and for which no primary citations were previously provided.

Units (µg/mL) and decimal formats should be standardized throughout.

**Authors response:** We thank the reviewer for pointing this out. All units (µg/mL) and decimal formats have now been standardized consistently throughout the manuscript to ensure clarity and uniformity.

In Section 2.7.5, the authors describe DFT optimization at the B3LYP level but do not report the basis set used or confirm that optimized geometries were minima by verifying the absence of imaginary frequencies. It is essential to ensure that the optimized structures correspond to true ground-state minima on the potential energy surface. The authors are suggested to report the number of imaginary frequencies (NImag = 0) for each structure and provide xyz coordinates or fchk files of the optimized geometries.

**Authors response:** Thank you reviewer, you are right, we didn’t mention the basses set , now it has been mentiond. The basis set 6-31G was used along with B3LYP, Only Geometry Optimization was performed and no frequencies were calculated. The chk files of optimized structure have been provided.

It is suggested that the below mentioned foundational sources would enrich and depth author's discussion on hardness, softness, chemical potential and reactivity based on DFT.

-Muya, Jules Tshishimbi, et al. "Conceptual DFT study of the chemical reactivity of four natural products with anti-sickling activity." SN Applied Sciences 1.11 (2019): 1457.

-Akintemi, Eric O., Krishna K. Govender, and Thishana Singh. "A DFT study of the chemical reactivity properties, spectroscopy and bioactivity scores of bioactive flavonols." Computational and Theoretical Chemistry 1210 (2022): 113658.

-Kour, Manjinder, Raakhi Gupta, and Raj K. Bansal. "Experimental and theoretical investigation of the reaction of secondary amines with maleic anhydride." Australian Journal of Chemistry 70.12 (2017): 1247-1253.

-Hussein, Y. T., & Azeez, Y. H. (2023). DFT analysis and in silico exploration of drug-likeness, toxicity prediction, bioactivity score, and chemical reactivity properties of the urolithins. Journal of Biomolecular Structure and Dynamics, 41(4), 1168-1177.

**Authors response:** Thank you for your valuable comment regarding the theoretical basis for our findings. To provide a robust quantum chemical rationale for the observed results, we have performed a detailed conceptual DFT analysis and more detailed has been added in manuscript.

Figures need higher resolution and consistent labeling of axes and units. Figures quality at present is very poor and not up to the standards.

**Authors response**: We appreciate the reviewer’s helpful feedback. All figures have been replaced with high-resolution versions, and axis labels, units, and legends have been standardized across all figure panels to comply with journal quality requirements.

**Reviewer #3**

Reviewer #3: General assessment:

This manuscript investigates the bioactive potential of an aqueous stem extract of Withania coagulans through GC–MS

2. Antibacterial and enzyme-inhibition data

o The agar-well diffusion assay alone is insufficient to establish antibacterial potency. Please provide MIC and MBC values using broth microdilution.

**Authors response**: Thank you for this important observation. In response, we have now conducted MIC and MBC assays using the broth microdilution method, following CLSI M07-A9 guidelines.

o For α-amylase inhibition, report dose–response curves and IC₅₀ values with clear replicate numbers, error bars, and statistical tests.

**Authors response:** We sincerely thank the reviewer for this valuable suggestion. In the revised version, we have included α‑amylase inhibition data at three different concentrations (10, 20, and 30 µg/mL) for both *Peganum harmala* and the standard drug acarbose. The results are presented as mean ± SD (n = 3).

We acknowledge that a complete IC_50_ determination would require a wider range of concentrations. Due to resource constraints, the current study was limited to three concentrations. However, we fully agree with the reviewer, and we are planning to conduct extended dose–response experiments to determine accurate IC_50_ values in future work. Thanks for understanding.

3. GC–MS identification confidence

o Specify library match scores, retention-index confirmation, and whether derivatization was applied.

**Authors response:** We thank the reviewer for this important methodological clarification. We have now explicitly stated in the Materials and Methods section (2.3 GC–MS analysis)

o Discuss the limitations of GC–MS for aqueous extracts and, if possible, confirm key compounds with HRMS or co-injection with standards.

**Authors response:** Thank you for your comment. We acknowledge that GC-MS has limitations with aqueous extracts in discussion section

4. Computational methods and reproducibility

o Several MD parameters appear inconsistent (e.g., very short equilibration, 0.00 M NaCl, unclear cut-offs). Provide full GROMACS parameter (.mdp) files, grid coordinates, and detailed docking parameters.

**Authors response:** Thank you for your comment. We have revised our computational methods, correcting the MD parameters for ionic strength, cut-offs, and implementing a more robust equilibration protocol. As requested, docking parameters have been added in manuscript, and the complete GROMACS .mdp files the Supplementary Information to ensure full reproducibility.

o Report how docking poses were validated (e.g., RMSD of redocked co-crystal ligands).

**Authors response:** Thank you for your suggestion. The co-crystal ligands were re-docked and then compared with the original docking pose and an RMSD of less than 2 Angstrom was observed which validated the docking protocol. Redocked Files have also been provided in raw data.

5. Interpretation and tone of claims

o Revise language to avoid causal overstatement (e.g., replace “are multi-target inhibitors” with “are predicted multi-target inhibitors present in the extract”).

**Authors response:** Thank you dear reviewer. We revised the manuscript.

o Differentiate clearly between experimental evidence and computational prediction.

**Authors response:**

Minor Comments

1. Clarify concentrations and volumes used in agar diffusion wells and enzyme assays.

**Authors response:** Thank you for your comment. We have revised the Materials and Methods sections to clearly specify both the concentrations

2. Report biological and technical replicate numbers for all assays and include the statistical methods used.

**Authors response:** Thank you for your comment. We have updated the Statistical Analysis section to include the details of the computational studies. All assays were performed with three biological replicates, each with three technical replicates

3. Expand figure legends to indicate sample size and significance levels.

**Authors response:** Dear Reviewer thank for you for your valuable comments , revised

4. Correct typographical errors and unit inconsistencies in the MD and results sections.

**Authors response:** Thank you for your comment: Revised thoroughly in the manuscript.

5. Ensure all software tools are cited with versions and references.

**Authors response:** Dear reviewer Thank you for your comments. As per your suggestion, we updated the whole manuscript by cited the tools with their version.

6. Include detailed step-by-step protocols for extraction, assays, and computational analyses as supplementary files.

**Authors response:** Thank you dear reviewer, added and attached

Recommendation

Decision: Major Revision (encouraged to resubmit)

The manuscript has merit and could make a meaningful contribution once the above issues, especially experimental validation of lead compounds, reproducibility of computational methods, and data deposition, are fully addressed.

**Reviewer #4**

Reviewer #4: The manuscript would benefit from several revisions to improve consistency, methodological clarity, and alignment with journal standards. Please standardize equation numbering and placement so that references in the text (e.g., “(Eq. 1)”) correspond unambiguously to right-aligned, sequentially labeled equations. In the Introduction, adopt a single citation style; for example, use bracketed, comma-separated numerals throughout rather than mixing semicolons and commas. Define deionized water at first mention as “deionized (DI) water” and use “DI water” consistently instead of “DW.”

**Authors response:** Thank you for this helpful suggestion. Equation numbering has been standardized and sequentially aligned with in-text references. Citation formatting in the Introduction has been revised to use a consistent bracketed numeral style. “Deionized (DI) water” is now defined at first mention, and “DI water” is used consistently throughout the manuscript.

In the GC–MS section, the current description of aqueous extract analysis does not address volatility of polar constituents; either include a nonpolar fractionation step prior to GC–MS or add a derivatization protocol (reagent, temperature, time, and any cleanup) suitable for the analytes reported. For the antibacterial assay, erythromycin at 30 µg is a weak comparator for Gram-negative organisms; replace or supplement it with a CLSI-concordant control for Gram-negatives (e.g., ciprofloxacin 5 µg disk, gentamicin 10 µg, or imipenem) and interpret results per CLSI M100. Verify the Salmonella ATCC designation and serovar (e.g., commonly S. Typhimurium ATCC 14028) and update all text and figure labels accordingly. Report diffusion assay units in the appropriate format—µg per disk for disk diffusion, or mass per well (µg) together with the loaded volume (µL) for well diffusion—and include inoculum standardization, agar depth, incubation conditions, and vehicle controls.

**Authors response:** Thank you for your feedback. We have updated thoroughly.

For the α-amylase assays, clarify the DNSA control definitions and the inhibition equation explicitly (A_control = enzyme + substrate without inhibitor; A_sample = enzyme + substrate + test item; % inhibition = [(A_control − A_sample)/A_control] × 100), and reconcile these definitions with what is plotted in the figures. The starch diffusion assay incubation of 72 h at 37 °C is atypically long for an enzyme plate and risks denaturation or contamination; shorten to a validated, substantially shorter period and justifying the chosen time. In the protein-preparation workflow, specify whether catalytically or structurally relevant crystallographic waters and cofactors within a defined distance of the binding site were retained, describe protonation state assignment at pH 7.0–7.4, and indicate how histidine tautomers were resolved.

**Authors response:** We appreciate the reviewer comments. Revised and in this study, all non-protein components—including crystallographic water molecules and cofactors were removed during the protein-preparation stage in order to obtain a purified protein structure for the Biovia Discovery workflow. Because our analysis did not rely on water-mediated or cofactor-dependent interactions, retaining these components was not required.

Finally, moderate structure–activity assertions to match analytical confidence. Replace unsupported statements assigning anticancer activity to compounds such as propylamine or methyl ethyl cyclopentene with conservative language (e.g., that certain terpenoids/phenolics have reported cytotoxic activities in the literature and that the present identifications are tentative and require validation). Remove attributions involving Dimefox from any therapeutic discussion or explicitly exclude such tentative matches from interpretation pending confirmation with authentic standards and retention indices. Implementing these revisions will improve reproducibility, interpretability, and compliance with field conventions.

**Authors response:** Thank you for your helpful comments. We have revised the manuscript to moderate structure–activity assertions and use more conservative language regarding the anticancer activity of compounds like propylamine and methyl ethyl cyclopentene. We have also clarified that Dimefox’s potential therapeutic activity is tentative and requires validation with authentic standards. These revisions have been made to improve the reproducibility and interpretability of the findings.

**Reviewer #5**

Reviewer #5: This manuscript titled “Identification of Two Terpenoids from *Withania coagulans* as Multi-Target Inhibitors: An In Vitro and In Silico Study” explores the pharmacological potential of *Withania coagulans* through a combination of experimental and computational methods. The topic is scientifically relevant and aligns with current interests in plant-based therapeutics and integrated computational validation. However, the manuscript requires substantial improvements in methodological detail, analytical rigor, and presentation to meet publication standards. The following comments address key technical and structural issues aimed at enhancing its clarity, reproducibility, and scientific quality.

Provide quantitative statistics for all biological assays (mean ± SD, ANOVA, or t-test results).

**Authors response:** Thank you for your comment. we have updated the manuscript to report mean ± standard deviation (SD) for all biological assays.

Include docking validation (redocking RMSD ≤ 2.0 Å) and specify detailed grid parameters.

**Authors response:** Thank you dear reviewer for valuable consideration. We have done it and redocked files are provided as pdb. format in raw data.

Expand ADMET results with full descriptors and cross-validate using at least one additional predictive platform.

**Authors response:** Thank you for comment. In accordance with the reviewer's recommendation, we have cross-validated the pharmacokinetic predictions for our two lead compounds (Caryophyllene oxide and 2,2-Dimethyl-3-(…)-oxirane) using pkCSM predictive platform. This provides an important comparison to our initial results obtained from SwissADME. The comparative data has been compiled and presented in a new supplementary table, Table 3S, in the revised manuscript. In table 2S other descriptors that usually documented in previous literature, like TPSA, CYP1A2 inhibitor ,Veber,Egan were also added.

Report MMGBSA energies numerically (ΔG_total, Evdw, Eele, Gsolv) and include confidence intervals.

**Authors response:** We thank the reviewer for emphasizing the importance of a detailed numerical presentation of the MM/GBSA results. We would like to clarify that this information is now updated in the manuscript as in table 4.

Clarify the DFT interpretation by distinguishing between stability (large energy gap) and reactivity (small energy gap).

**Authors response:** We thank the reviewer for this excellent suggestion to improve the clarity of our DFT analysis. We agree that a more explicit distinction between molecular stability and reactivity is crucial for a robust interpretation of the results.

Enhance reproducibility by including molecular simulation input parameters and convergence criteria.

**Authors response:** Thank you for your consideration. The MD parameters in material method section has been updated as per your suggestion in the manuscript.

Correlate computational outputs with biochemical validation (e.g., enzyme inhibition constants) to strengthen the mechanistic foundation.

**Authors response:** Thank you for this valuable suggestion. In response, we have revised the Results and Discussion sections to better correlate the computational findings (e.g., docking scores, MD simulations, DFT, ADMET) with the experimental enzyme inhibition data from the α-amylase assay.

**Comments to editor**

Dear Editor,

We are grateful for the opportunity to revise our manuscript. We have carefully addressed all the comments and suggestions provided by the reviewers.

In our point-by-point response document:

The responses to reviewer comments are presented in **red font** for clarity and grammatical improvements are highlighted in **blue text.**

All necessary changes have been incorporated into the revised manuscript accordingly. We sincerely thank you and the reviewers for your constructive feedback, which has greatly improved the quality of our work. We hope the revised version meets the standards for publication in PLOS ONE.
