# Supplementary material for: Identification of two terpenoids from Withania coagulans with predicted multitarget binding affinity: An in vitro and in silico study
Source: PLoS One. 2026 Feb 20;21(2):e0343273. doi: 10.1371/journal.pone.0343273 (PMC12923132; doi:10.1371/journal.pone.0343273)
Supplement: Attachment — Submitted filename: Point by point response plos one R2.docx [file pone.0343273.s009.docx]

**AHMED VANDY**

[ahmedvandy@gmail.com](mailto:ahmedvandy@gmail.com) / +232-79-522721

[LinkedIn](https://www.linkedin.com/in/ahmed-vandy-964201159/) / [ORCID](https://orcid.org/0009-0008-2603-9989)

29^th^ January, 2026.

Dear Editor and the Reviewers,

We would like to express our sincere gratitude for your constructive feedback and for the time you dedicated to reviewing our manuscript titled **"** **Identification of two terpenoids from Withania coagulans with predicted multitarget binding affinity: An in vitro and in silico study"**. We deeply appreciate your thoughtful and insightful comments, which have significantly improved the quality of our submission.

**Additional Editor Comments:**

Understandably, the authors have responded positively to the previous concerns. The revision has improved the quality of the submission significantly. However, some major points still deserve a substantial attention of the authors as specifically pointed out by Reviewer #1. I hereby recommend another round of revision to address these.

**Authors response:** Thank you for your constructive feedback and for recognizing the improvements made in the revised manuscript. We sincerely appreciate the time and effort you and Reviewer #1 have dedicated to reviewing our work.

Unarguably, we agree to the points highlighted by Reviewer #1. We want to assure you that we have carefully reviewed these concerns and have made the necessary revisions to address them in detail. All feedback has been thoroughly incorporated into the manuscript, and we believe the updated version now meets the expectations of the reviewers.

We appreciate your recommendation for another round of revision and are confident that the changes made will significantly improve the quality of the submission.

Thank you once again for your guidance. We look forward to your further feedback.

**Reviewers' comments:**

Please use the space provided to explain your answers to the questions above. You may also include additional comments for the author, including concerns about dual publication, research ethics, or publication ethics. (Please upload your review as an attachment if it exceeds 20,000 characters)

**Reviewer #1:**

The authors present a multidisciplinary study integrating phytochemical profiling (GC–MS), in vitro biological assays (antibacterial, antidiabetic, antioxidant), and extensive computational validation (docking, ADMET, DFT, MD simulations, MM/GBSA) to investigate the pharmacological potential of Withania coagulans stem extract, with a focus on two terpenoids. The revised submission addresses most of the major and minor concerns raised in the previous round of review, resulting in a significant improvement in both scientific rigor and presentation.

Provided the following edits and clarifications are made, it will be suitable for publication-

1) While the integration is new for W. coagulans, similar strategies have been applied to other medicinal plants, making the contribution incremental rather than transformative. In the introduction, explicitly highlight how this work advances beyond previous studies on W. coagulans and terpenoids.

**Authors response**; Thank you for your constructive feedback. In response to your comment regarding the novelty of the study, we have revised the introduction to explicitly highlight how this work advances beyond previous studies on *Withania coagulans*. In this study, for the first time, we explore terpenoid compounds in *W. coagulans* beyond the well-known withanolides, revealing a new dimension of the plant’s medicinal potential. This approach uncovers previously unreported terpenoids and their multi-functional properties, particularly their promising applications in anticancer strategies. These points have been added and revised in the introduction to emphasize the novelty and significance of our findings.

2) The manuscript could better articulate how its findings compare to existing therapeutics and the broader implications for drug development. Expand the discussion to compare findings with current antimicrobial agents and discuss the potential for clinical translation.

**Authors response**; Thank you for your valuable feedback. In response to your suggestion, we have expanded the Discussion section to better articulate how our findings compare to existing antimicrobial agents and their broader implications for drug development.

3) The methods section lacks critical detail, especially for the antimicrobial assays, making it difficult to reproduce the work.

**Authors response**; Thank you for your valuable feedback regarding the methods section. In response, we have revised and updated the methodology to provide more detailed and reproducible instructions, particularly for the antibacterial activity assay.

4) Table 3 omits standard deviations for zone of inhibition measurements, despite the legend stating data are presented as mean ± SD. Apply appropriate statistical tests, and present results as mean ± standard deviation with corresponding p-values.

**Authors response**; Thank you for your constructive feedback regarding Table 3 and the inclusion of standard deviations (SD) and statistical analysis. In response to your comment, we have updated Table 3 to include the standard deviations (SD) for the zone of inhibition measurements and the corresponding p-values for statistical significance.

5) The manuscript does not describe the use of standard antibiotics (e.g., erythromycin) in the MIC and MBC assays. Include MIC and MBC values for these standards in Table 4 to enable direct comparison with test compounds. Apply appropriate statistical tests and report all data as mean ± standard deviation with p-values.

**Authors response**; Thank you for your valuable feedback. In response to your comment, we have now included the MIC and MBC values for erythromycin (a standard antibiotic) in Table 4, allowing for a direct comparison with the test compounds, such as *Withania coagulans* extract.

**Reviewer #2:**

While all the reviewers comments has been addressed, there are still some formatting improvements pending mainly consistency. For example, ΔE_gap versus ΔE_Gap, ΔG_TOTAL versus ΔGTOTAL and so on. Please make sure to make these things carefully consistent while proofreading.
 **Authors response**; Thank you dear reviewer: Thoroughly revised and updated.

**Reviewer #3:**

The article strength lies in its transparent and methodologically rigorous integration of phytochemical profiling, in vitro biological screening, and validated in silico analyses, including molecular docking, molecular dynamics simulations, MM/GBSA binding energetics, and DFT calculations. The manuscript demonstrates strong reproducibility through detailed protocol reporting, validated computational workflows, and full data availability in compliance with PLOS ONE standards. By focusing on underexplored terpenoid constituents of Withania coagulans, the work provides a sound, hypothesis-generating contribution to medicinal-plant research while maintaining appropriate interpretive caution.
 **Authors response**; Thank you for your positive feedback. We appreciate your recognition of the rigorous integration of phytochemical profiling, in vitro screening, and in silico analyses in our study. We are also glad that you found our focus on underexplored terpenoids in *Withania coagulans* to be a valuable contribution to medicinal-plant research. Your encouraging comments are greatly appreciated.

**Reviewer #4:**

The English used is very much correct. but i believe the author should cross check all to identify where there is a typographiccal error and correct it all.

**Authors response**; Thank you dear reviewer: Thoroughly revised and updated.

**Comments to editor:** We have carefully addressed all the reviewer comments and made the necessary revisions to the manuscript. The changes are clearly marked in red text, and blue text highlights the grammatical corrections. We believe these revisions have strengthened the manuscript, and we are confident that it now meets the publication standards. We appreciate your continued guidance and look forward to your further feedback.
