# Supplementary material for: Identification of two terpenoids from Withania coagulans with predicted multitarget binding affinity: An in vitro and in silico study
Source: PLoS One. 2026 Feb 20;21(2):e0343273. doi: 10.1371/journal.pone.0343273 (PMC12923132; doi:10.1371/journal.pone.0343273)
Supplement: Attachment — Submitted filename: Reviewer comments.docx [file pone.0343273.s006.docx]

The integration of phytochemical profiling, biological assays, and computational modeling addresses a relevant topic in medicinal-plant research. The authors attempt to connect *in-vitro* results with *in-silico* validation. The selection of *Withania coagulans*, a well-known ethnomedicinal species, is of genuine pharmacological interest and aligns with the journal’s interdisciplinary scope.

Following are my specific comments for the authors:

The extraction procedure is well described, but the authors should report the extraction yield (% w/w), specify the working extract concentration, and briefly justify the choice of water as the solvent. This information is helpful for reproducibility and comparison with earlier studies.

The inclusion of at least one Gram-positive strain is suggested (not required though). But the inhibition zones data should be presented with means ± SD from triplicates and statistically compared to the control.

The starch hydrolysis assay based on inhibition zone measurement is unconventional. Please add brief rationale and citation.

The ADMET table gives nearly identical results for all compounds despite their structural diversity? A comment.

The paper repeatedly describes the two terpenoids as “multi-target inhibitors” and “potential drug candidates.” One would be more cautious as the study is limited to preliminary in-vitro assays and in-silico predictions.

The discussion also includes statements, for example, assigning cardiovascular or anticancer properties to compounds that were neither tested nor cited from primary literature. Wherever possible, the authors should avoid extrapolations beyond the evidence.

Units (µg/mL) and decimal formats should be standardized throughout.

In Section 2.7.5, the authors describe DFT optimization at the B3LYP level but do not report the basis set used or confirm that optimized geometries were minima by verifying the absence of imaginary frequencies. It is essential to ensure that the optimized structures correspond to true ground-state minima on the potential energy surface. The authors are suggested to report the number of imaginary frequencies (NImag = 0) for each structure and provide xyz coordinates or fchk files of the optimized geometries.

It is suggested that the below mentioned foundational sources would enrich and depth author's discussion on hardness, softness, chemical potential and reactivity based on DFT.

-Muya, Jules Tshishimbi, et al. "Conceptual DFT study of the chemical reactivity of four natural products with anti-sickling activity." *SN Applied Sciences* 1.11 (2019): 1457.

-Akintemi, Eric O., Krishna K. Govender, and Thishana Singh. "A DFT study of the chemical reactivity properties, spectroscopy and bioactivity scores of bioactive flavonols." *Computational and Theoretical Chemistry* 1210 (2022): 113658.

-Kour, Manjinder, Raakhi Gupta, and Raj K. Bansal. "Experimental and theoretical investigation of the reaction of secondary amines with maleic anhydride." *Australian Journal of Chemistry* 70.12 (2017): 1247-1253.

-Hussein, Y. T., & Azeez, Y. H. (2023). DFT analysis and in silico exploration of drug-likeness, toxicity prediction, bioactivity score, and chemical reactivity properties of the urolithins. *Journal of Biomolecular Structure and Dynamics*, *41*(4), 1168-1177.

Figures need higher resolution and consistent labeling of axes and units. Figures quality at present is very poor and not up to the standards.

Once these issues are addressed, the work could make a useful contribution to the literature on *Withania coagulans* phytochemistry and bioactivity.
